# MENINGES HARBOR IMMUNE MEMORIES OF LIFE EXPERIENCES

**DOI:** 10.1093/geroni/igac059.1373

**Published:** 2022-12-20

**Authors:** Jasmin Herz, Khalil Alves de Lima, Andrea Francesca Salvador, Mackenzie Lemieux, Taitea Dykstra, Igor Smirnov, Jonathan Kipnis

**Affiliations:** Washington University in St. Louis, School of Medicine, St. Louis, Missouri, United States; Washington University in St. Louis, St. Louis, Missouri, United States; Washington University in St. Louis, St. Louis, Missouri, United States; Washington University in St. Louis, St. Louis, Missouri, United States; Washington University in St. Louis, St. Louis, Missouri, United States; Washington University in St. Louis, St. Louis, Missouri, United States; Washington University in St. Louis, School of Medicine, St. Louis, Missouri, United States

## Abstract

The adaptive immune system relies on formation of the memory of past microbial challenges to accelerate protective immune responses in the event of reinfection. This memory is accomplished in part by the retention of antigen-specific B and T cells within barrier tissues. As the healthy central nervous system parenchyma is virtually devoid of adaptive immune cells, the meningeal spaces carry out the vital function of coping with environmental threats during aging. We conducted an extensive molecular and functional analysis of meningeal T cells to test the hypothesis that the meninges in the brain sense and respond to internal and external cues throughout life, and that alterations in the meningeal T cell repertoire alter brain function. We found presumably self-reactive tissue-resident T cells in the meninges of naive mice. Using models of pathogen exposure, we describe a neuroimmune axis in which antigen experienced resident Tcell subsets dynamically record immune perturbations, which resulted in behavioral abnormalities that were exacerbated with aging. Our findings elucidate molecular properties of T cells that survey the brain borders under both homeostatic and pathological conditions and provide insights linking CNS immune privilege with memory.

